# Anti-Drug Antibodies: Emerging Approaches to Predict, Reduce or Reverse Biotherapeutic Immunogenicity

**DOI:** 10.3390/antib7020019

**Published:** 2018-05-31

**Authors:** Kathleen P. Pratt

**Affiliations:** Department of Medicine (MED) A3075, Uniformed Services University of the Health Sciences, 4301 Jones Bridge Road, Bethesda, MD 20814, USA; Kathleen.pratt@usuhs.edu; Tel.: +1-301-295-3607; Fax: +1-301-295-3557

**Keywords:** anti-drug antibodies, biotherapeutics, immunogenicity, tolerance

## Abstract

The development of anti-drug antibodies (ADAs) following administration of biotherapeutics to patients is a vexing problem that is attracting increasing attention from pharmaceutical and biotechnology companies. This serious clinical problem is also spawning creative research into novel approaches to predict, avoid, and in some cases even reverse such deleterious immune responses. CD4^+^ T cells are essential players in the development of most ADAs, while memory B-cell and long-lived plasma cells amplify and maintain these responses. This review summarizes methods to predict and experimentally identify T-cell and B-cell epitopes in therapeutic proteins, with a particular focus on blood coagulation factor VIII (FVIII), whose immunogenicity is clinically significant and is the subject of intensive current research. Methods to phenotype ADA responses in humans are described, including T-cell stimulation assays, and both established and novel approaches to determine the titers, epitopes and isotypes of the ADAs themselves. Although rational protein engineering can reduce the immunogenicity of many biotherapeutics, complementary, novel approaches to induce specific tolerance, especially during initial exposures, are expected to play significant roles in future efforts to reduce or reverse these unwanted immune responses.

## 1. Introduction

The administration of biological (e.g., protein) drugs to patients, especially serial exposures to treat chronic conditions, carries a risk of eliciting anti-drug antibodies (ADAs) [1,2,3]. This risk is higher if the biotherapeutic is a ‘non-self’ substance, e.g., a protein replacement therapy to treat monogenic diseases such as severe hemophilia A [4] or B [5], Pompe’s disease [6], etc. However, even monoclonal antibodies, which comprise the largest class of therapeutic proteins, can provoke ADAs in some patients [7,8,9]. For example, the immunogenicity of anti-tumor necrosis factor (TNF) α antibodies used to treat rheumatoid arthritis, and of biotherapeutics prescribed to alleviate symptoms of inflammatory bowel disease (IBD), is a significant problem, compounded by the inflammatory milieu of the clinical disorders requiring treatment [10,11,12]. The development of IgE antibodies can provoke serious hypersensitivity, as was seen in immune responses to the galactose-α-1,3-galactose moiety on cetuximab; this problem was alleviated by utilizing a Chinese Hamster Ovary (CHO) expression system that did not perform this post-translational modification [13].

Neutralizing ADAs negate the clinical benefit of the biotherapeutic agent, while non-neutralizing antibodies can also reduce efficacy, e.g., by affecting clearance, pharmacodynamics and pharmacokinetics. Immune responses to therapeutic proteins that are “biosimilars” to endogenous proteins, or even having identical amino acid sequences to endogenous proteins, may provoke antibodies that bind to both the endogenous and the therapeutic protein; such off-target effects could be serious, and even lethal, if the endogenous protein performs an essential function. To give a recent example, recombinant activated factor VII (rFVIIa) is an endogenous serine protease that initiates the extrinsic blood coagulation cascade, and it is an alternative “bypass” treatment for hemophilia A or B patients who have become non-responders to their replacement therapy (usually due to development of ADAs against infused factor VIII (FVIII) or factor IX (FIX), respectively). It also has been used extensively off-label to treat hemorrhagic bleeds, e.g., following trauma. A rationally designed rFVIIa analog having higher enzymatic activity due to only three amino acid substitutions, compared to the wild-type sequence, was developed and tested for efficacy in hemophilia A and B patients. Despite the 99% sequence identity of the ‘improved’ rFVIIa with wild-type (endogenous) FVIIa, eight of 72 patients enrolled in a phase III clinical trial developed antibodies against the biotherapeutic, and four of these polyclonal responses showed cross-reactivity to the wild-type FVIIa protein [14]. This strong immunogenicity had not been anticipated, as the wild-type protein had been in clinical use for over 20 years with no reports of ADA responses [15,16,17]. Fortunately, the antibodies were detected and the trial halted before titers increased to significantly impact hemostasis. Another sequence-modified rFVIIa variant was tested in a separate trial, and the immune response of one patient who developed ADAs evaluated; interestingly, subsequent epitope mapping indicated that this subject’s PBMCs responded to synthetic peptides having the wild-type FVIIa sequence, but not to peptides corresponding to the modified amino acid sequence [18]. Therapeutic administration of some endogenous proteins, e.g., erythropoietin (Epo) [19,20] and interferon-β [21,22], has been shown to provoke unwanted, rare, but potentially serious immunogenicity. In some cases anti-Epo antibodies may provoke antibody-mediated red cell aplasia [23,24]. The development of more effective methods to predict and reduce protein immunogenicity during product development, rather than encountering immune responses in the course of expensive clinical trials, is a compelling priority to improve effectiveness and safety profiles of biotherapeutics.

This review will first briefly summarize the etiology of ADA development, including roles of T-cell and B-cell responses to foreign antigens, and in some cases the pre-existence of ADAs in naïve subjects. *In silico* and experimental methods to predict and confirm T-cell and B-cell epitopes will then be described, as will approaches to “de-immunize” proteins through rational amino acid substitutions and/or attachment of epitope-masking moieties to biotherapeutics. Several cases where the successful application of such techniques has demonstrably reduced protein immunogenicity will be summarized, along with caveats. Analytical methods to profile the ADAs themselves include epitope mapping and quantification of total specific antibody titers and isotypes/subclasses. Rather than tempering enthusiasm for the translational potential of protein drugs, addressing the remaining challenges to reduce their immunogenicity will require creative approaches that are certain to improve our understanding of human immunology, and in particular the mechanisms by which immune tolerance to specific antigens may be achieved or restored.

## 2. Most ADAs Require CD4^+^ T-Effector Help

Multiple exposures to a foreign antigen, especially in context of inflammation or innate immune signaling (“danger”), can prime and boost the host immune response, resulting in production of high-affinity, high-titer, class-switched antibodies. This essential defense mechanism, however, is an unwanted or even adverse event when activated in response to a biotherapeutic intended to treat a clinical disorder. The situation is even more complex if a biotherapeutic is a sequence-modified version of a self-protein, a large category that includes therapeutic monoclonal antibodies, as memory effector and regulatory T and B cells may be stimulated and/or exhibit plasticity that will be deeply conditioned by the local environment in which antigen presentation occurs. Hence, even patients with similar HLA-restricted recognition of epitopes in the same therapeutic protein may have significantly different immune outcomes (anti-drug antibodies, CD8^+^ T-effector responses, or tolerance).

Biotherapeutics constitute a large and growing segment of pharmaceutical company portfolios, and their early development is also powered by significant investments from governments and private foundations. Over the past ~20 years, as more biologic drugs have progressed through preclinical testing into clinical trials, realization has grown that immunogenicity should be considered an expected outcome, rather than a possible complication, and that potential immunogenicity should be tested and addressed early in the translation from bench to bedside.

Therapeutic proteins, whether endogenous or “non-self”, may be endocytosed by antigen-presenting cells (APCs) following administration, where they are processed in the immunoproteasome to their component peptides. A subset of these peptides may then be presented on an individual’s Major Histocompatability Complex (MHC), restricted to their HLA Class I and/or Class II alleles [25,26]. Importantly, this initial presentation may lead to either tolerogenic or T-effector responses providing help for B cells in germinal centers and/or activating cytotoxic T-lymphocytes (CTLs). We focus here on the roles of CD4^+^ T cells in T-effector antibody help versus tolerance, but the CTL response is a related potential concern that should also be considered early in the drug design process.

Dendritic cells (DCs) are professional APCs whose role in amplifying antigen-specific immune responses has been appreciated for decades [27,28]. In addition to recognizing and processing specific antigen they can also detect damage-or pathogen-associated molecular pattern molecules through toll-like receptors on their surface, activating innate immune pathways [29]. The coordination of innate and adaptive immunity by DCs proceeds through up-regulation of MHC Class II (MHCII) and CD80 surface expression, thereby lowering the threshold for activation of naïve T cells that can recognize a specific peptide presented on MHCII (signal 1). Engagement of CD80/86 with CD28 on T cells generates co-stimulatory signaling (signal 2). Further T-cell stimulation with inflammatory cytokines generates signal 3, promoting T-effector proliferation and secretion of cytokines and chemokines, especially in germinal centers where B cells are then activated and proceed to differentiate into antibody-secreting plasma cells and memory B cells.

## 3. Potential of Tolerogenic DC Presentation to Prevent ADAs

In the absence of co-stimulation, T cells may become anergic. Immature, tolerogenic DCs (iDCs) are a distinct DC subset that can efficiently present peptides without promoting strong co-stimulation of naïve T cells; iDCs secrete low levels of interleukin (IL)-12 and higher levels of IL-10 and TGF-β and are thought to contribute to tolerance via anergy and deletion of T-effectors, as well as clonal stimulation of antigen-specific CD4^+^CD25^+^FoxP3^+^ T-regulatory cells (Tregs) [30]. IDCs migrate through the periphery and lymph, where they play an important role in maintaining tolerance to self-antigens via suppression of auto-reactive T-effectors. Their migratory nature and promotion of antigen-specific tolerance suggest that avoidance of “danger” signals during initial exposures to a biotherapeutic drug may allow clinicians to capture the potential of iDCs to facilitate peripheral drug-specific tolerance. Interestingly, durable tolerance to self-antigens is promoted by iDCs following their engulfment of apoptotic cells, but not necrotic cells, thereby maintaining tolerance to intracellular antigens that become exposed to the immune system during normal cellular turnover in the absence of danger signals [31]. The potential of cellular therapies employing apoptotic depletion of T cells to decrease allograft rejection is being explored [31], and ex vivo skewing of monocyte-derived DCs to a tolerogenic phenotype [32] holds promise for iDC-based therapies.

The antigenic landscape of specific biotherapeutics (e.g., recombinant proteins) is considerably less complex than the immune challenges accompanying organ transplantation or autoimmunity. Therefore, additional, alternative or complementary approaches to present biotherapeutic-derived peptides on tolerogenic iDCs can be considered, including manipulation of the antigen itself to reduce peptide-MHCII presentation on the surface of APCs. Such protein engineering to effectively reduce immunogenicity/antigenicity should be feasible due to involvement of far fewer immunodominant epitopes in provoking alloimmune responses. Indeed, the prediction, experimental validation, and alteration of CD4^+^ T-cell epitopes are increasingly being incorporated into the workflow in the design of novel biotherapeutics [3].

## 4. Identifying and Modifying HLA-Restricted T-Cell Epitopes in Biotherapeutics

The successful identification and removal of immunodominant CD4^+^ T-cell epitopes could clearly reduce the incidence and severity of ADAs through immunologic ignorance, if the peptide epitope is no longer presented on an individual’s MHCII. Alternatively, reduced MHCII affinity could lessen the probability of effective MHCII-peptide-TCR synapse formation leading to T-effector activation, proliferation and signaling, and could also promote Treg rather than T-effector expansion. Furthermore, epitope “de-immunization” through sequence modification of recombinant proteins is an attractive means of reducing their immunogenicity, as it occurs during the design phase of the biotherapeutic itself, rather than subsequently through clinical interventions during or after administration to patients.

In 2005, Sette and colleagues hypothesized that protein drug immunogenicity was due to T-cell responses to a limited number of immunodominant T-cell epitopes, which typically bound promiscuously to multiple HLA alleles [33]. This hypothesis was tested systematically for five therapeutic proteins that had demonstrated immunogenicity following clinical testing. Methods employed included *in silico* predictions of HLA-binding sequences and in vitro experiments testing binding affinities of synthetic, overlapping peptides for recombinant HLA-DR molecules. The immunogenicity of peptides spanning the amino acid sequence of erythropoietin (Epo) was also evaluated using ELISPOT assays of PBMCs from seven HLA-typed donors. Five of these donor PBMC samples secreted IL-2 in response to Epo peptides, and two immunogenic “hot spots” (residues 91–120 and 126–155) elicited the most frequent responses. Interestingly, similar ex vivo immunogenicity has also been demonstrated against other self-proteins [1,34,35,36], particularly if supra-physiological concentrations of the protein or peptide antigen are used to stimulate PBMCs or CD4^+^ T-cell fractions cultured with antigen-presenting cells, illustrating the fact that some auto-reactive, lower-avidity T cells escape thymic deletion and pose potential risks for future autoimmunity. Finally, sequence modification of Epo residues predicted to occupy anchor position P1 for the respective HLA-DRs had the expected effect of reducing binding affinities and immunogenicity [33]. This overall “de-immunization” strategy has been further refined and is now available on the Immune Epitope Database website [37]. Additional *in silico* algorithms have been developed, in the public and private sectors, to predict MHCII binding and T-cell epitopes [38,39,40,41,42], including epitopes that may have differential effects on Tregs versus T-effectors, referred to as “Tregitopes” [43,44] and algorithms that incorporate protein structural information to predict effects of amino acid substitutions on stability [41,45,46]. Similar T-cell proliferation assays and cytokine secretion assays, e.g., ELISPOTs, following stimulation with proteins and/or pooled or individual peptides are widely used to map clinically relevant T-cell epitopes in vaccines as well as biotherapeutics. The risk of immunogenicity of therapeutic monoclonal antibodies has been evaluated using in vitro assays utilizing PBMCs from naïve, healthy human donors to detect T-cell proliferation, cytokine secretion, etc. following stimulation with the biotherapeutic; such assays can also be useful in evaluating lot-to-lot variations in the biotherapeutic that affect immunogenicity, e.g., aggregation, contamination, glycosylation, or post-translational modifications [47,48].

Neutralizing ADAs are a particularly serious problem in hemophilia A (HA), an X-linked bleeding disorder resulting from lack of the circulating blood coagulation protein factor VIII (FVIII). Up to 1 in 3 severe HA patients develops neutralizing ADAs, referred to by hematologists as “inhibitors”, and even patients with mild or moderate HA resulting from missense mutations may develop an inhibitor [49]. In the latter case, CD4^+^ T-cell epitopes corresponding to the missense substitution site have been conclusively identified in several subjects by isolation of T-cell clones responding to peptides with the wild-type sequence at the missense site [50,51,52,53,54]. FVIII epitopes have also been identified and modified in animal model studies [55,56,57]. The number of epitopes contributing to ADAs in severe HA subjects is a subject of current research, and is relevant to protein “de-immunization” strategies.

Mapping of clinically relevant HLA-restricted T-cell epitopes benefits from combinations of experimental and *in silico* methods such as those mentioned above. For example, monocyte-derived dendritic cells (mo-DCs) may be expanded from HLA-typed donors, pulsed with a biotherapeutic, the HLA-peptide complexes pulled down using appropriate antibodies, and the presented peptides eluted and identified by mass spectrometry. The Voorberg laboratory has demonstrated that the presented FVIII peptides comprise only a subset of those that could potentially bind to specific HLA-DR, DP and DQ (as indicated by prediction algorithms and direct protein-peptide binding experiments [58,59,60]). Similar methods have been used to map autoimmune epitopes in the blood protein ADAMTS13 [61]. *In silico* algorithms may then be employed to predict the MHC-binding registers within these presented peptides. T-cell proliferation or ELISPOT assays, or MHC tetramer staining, may also be carried out using synthetic peptides designed on the basis of binding and *in silico* experiments, to identify clinically relevant epitopes. Importantly, the peptides presented on specific HLA may be determined using blood samples from healthy controls, as well as recombinant HLA proteins and synthetic peptides, allowing more efficient T-cell stimulation experiments (requiring fewer peptides and less blood) to be carried out later with precious patient samples.

Our laboratory utilized MHCII tetramers loaded with FVIII peptides to query the T-cell response of a severe HA subject with an *F8* gene deletion mutation and a persistent high-titer inhibitor [62]. Systematic tetramer-guided epitope mapping is a daunting problem when applied to the FVIII protein due to its large size (2332 amino acid residues) and hence the need for large blood volumes to test overlapping peptides spanning the entire sequence. In this case, sufficient blood was donated to allow mapping of the known immunogenic FVIII A2, C1 and C2 domains. Interestingly, only one epitope was identified by tetramers, and T-cell receptor (TCR) beta variable sequencing of clones and polyclonal lines specific for this epitope indicated the TCR repertoire was highly oligoclonal. Although additional epitopes in the untested FVIII domains, as well as lower-avidity epitopes not identifiable by tetramers, may have also contributed to this immune response, these results provide encouragement for protein engineering attempts to reduce the immunogenicity of even large proteins such as FVIII. Indeed, strategic amino acid substitutions in this FVIII epitope at MHCII anchor positions P1 and P4 abrogated proliferation of the patient-derived clones and lines without compromising the protein’s specific activity [63]. The observed narrow epitope and TCR repertoire documented in this subject suggest that clonal deletion and anergy contribute to peripheral tolerance, although even some successfully tolerized patients continue to circulate FVIII-specific T-effectors [64], with their tolerance likely due to Treg dampening of the immune response [51]. Although not yet tested in the clinic, we hypothesize that removal of immunodominant CD4^+^ T-cell epitopes through FVIII protein engineering could lessen the inhibitor incidence in naïve HA patients and also improve success rates of clinical protocols to induce tolerance following inhibitor formation in multiply-infused patients.

Epitope de-immunization strategies have shown promise in reducing the immunogenicity of other sequence-modified proteins including recombinant immunotoxins used for cancer therapies [65,66,67,68,69,70,71,72], interferon-β [73], interferon-α [72], staphylokinase [74], β-lactamase [75], Epo [33,37], and monoclonal antibodies [37,76]. Admittedly, some immunodominant epitopes will correspond to sequences that cannot be altered without impacting functionality of the biotherapeutic, and the major impact of epitope mapping may eventually be to facilitate novel approaches to tolerize patients to specific antigens [77,78,79]. Protein “de-immunization” strategies and therapies to tolerize patients to specific antigens are highly complementary approaches that, if employed together, should synergize to improve patient outcomes. Protein engineering and process control improvements to reduce aggregation propensity can also reduce potential immunogenicity [80].

## 5. Identifying and Modifying B-Cell Epitopes in Biotherapeutics

B-cell epitopes are both antibody-binding surfaces and interfaces through which memory B cells become activated during recall responses. In addition, splenic marginal zone B cells and macrophages serve as sentinel messengers that can encounter biotherapeutics shortly after their administration, shuttling them (in some cases) to germinal centers where the naïve immune response is initiated [81,82,83,84], and where somatic hypermutation generates B cells with increasing affinity for the stimulating antigen [85]. Goals of B-cell epitope modification, therefore, include generating a biotherapeutic that can evade neutralization by pre-existing antibodies and/or reducing or abrogating signaling through B-cell receptors. B-cell epitopes may be mapped at high resolution by determining crystal structures of antigen-antibody complexes, and by hydrogen-deuterium (H/D) exchange/mass spectrometry to identify antigen-antibody interfaces that are protected from solvent [86,87], and hence from H/D exchange. Lower-resolution epitope mapping can be achieved by competition ELISA assays to analyze either MAbs [88] or polyclonal antibodies in plasma or serum [89,90,91].

**Figure 1:**
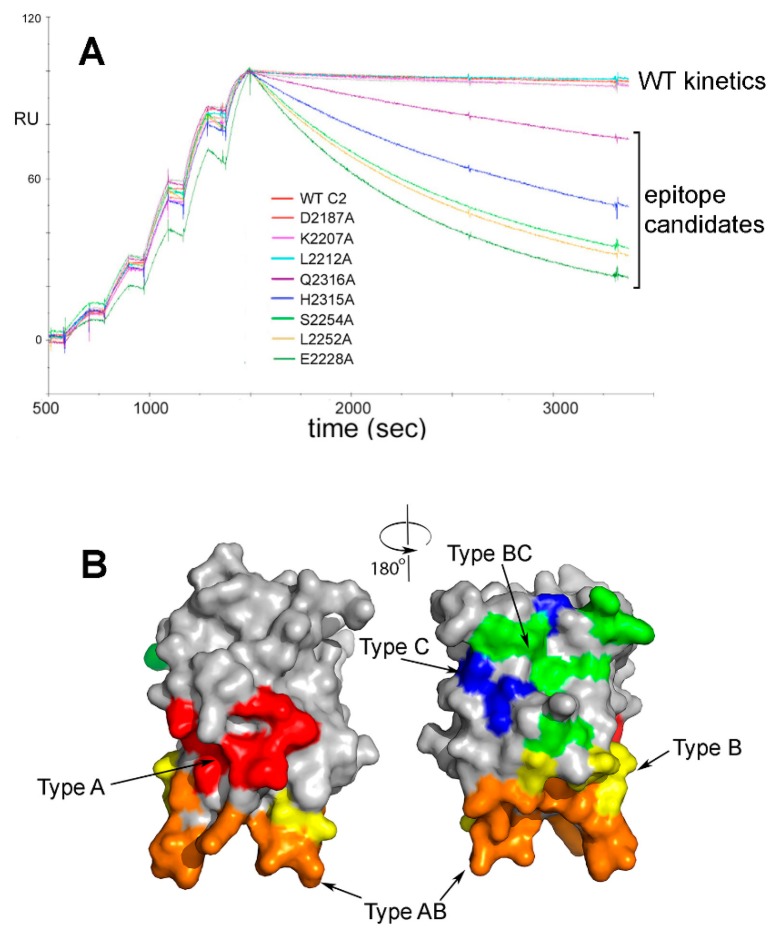
Surface plasmon resonance (SPR)-based epitope mapping. (**A**). Representative superimposed sensorgrams showing single-cycle kinetics experiments in which rFVIII-C2 protein and rFVIII-C2 variants with a single surface-exposed side chain mutated to alanine were injected at increasing concentrations over a FVIII-specific MAb captured on a biosensor [97]. Residues were flagged as potential contributors to the epitope if the k_d_ for the FVIII-C2 mutein was >2.0X the k_d_ for the wild-type rFVIII-C2 protein. Alanine substitutions at residues E2228, L2252, S2254, H2315 and Q2316 met this criterion in this set of experiments with MAb 1B5. Separate SPR runs (not shown here) identified residues F2196, T2197, N2198, F2200, T2202, R2220, Q2222, N2225 and K2239 as also possibly contributing to the epitope recognized by this MAb. (**B**). Front and back views (rotated 180°) of the FVIII C2 domain crystal structure [100], with surfaces colored to indicate the 5 partially-overlapping B-cell epitopes recognized by 11 neutralizing MAbs. The MAbs and their cognate epitopes were designated Types A, AB, B, BC and C on the basis of competition ELISA experiments. These MAbs inhibit distinct binding interactions and functions of FVIII, e.g., binding to negatively charged phospholipid surfaces, von Willebrand factor, and other proteins comprising the ‘intrinsic tenase’ complex [88]. Type A: red; Type AB: orange; Type B: yellow; Type BC: green; Type C: blue.

In the case of anti-FVIII ADAs, it has long been known that porcine FVIII can restore hemostasis in many HA inhibitor patients, although a subset of these patients eventually develop antibodies that neutralize porcine as well as human FVIII [92,93]. Building on this observation, rational modifications of B-cell epitopes in human FVIII are being pursued by several labs, including generation of hybrid human-porcine FVIII proteins [94,95]. Our laboratory approached B-cell epitope engineering of FVIII by first fine-mapping the epitopes recognized by neutralizing antibodies against its C-terminal C2 domain [96,97], a frequent target of neutralizing anti-FVIII ADAs [98,99]. First, 60 recombinant (r)FVIII-C2 domain proteins were generated, each with a single surface-exposed side chain mutated to alanine. Binding of these mutant proteins to a panel of neutralizing anti-FVIII MAb [88] was then evaluated by surface plasmon resonance (SPR) to measure the effects of these single-amino-acid changes on affinities [96,97]. These experiments identified a small set of residues comprising each “minimal epitope”, defined as the residues contributing the most to antibody affinity (Figure 1).

In 1995, Clackson and Wells described “hot spots” within larger protein-protein binding interfaces that contribute most of the binding affinity [101]. Use of this concept in B-cell epitope mapping and “de-immunization” strategies is attractive, because it limits the total number of amino acid substitutions required to generate less antigenic rFVIII proteins, thereby decreasing the probability of inadvertently introducing neo-epitopes. The SPR-identified epitopes were consistent with co-crystallization and other methods to characterize several of these antigen-antibody complexes [86,102,103,104], thereby further validating the method.

In other applications, the Pastan lab has modified B-cell epitopes of recombinant immunotoxins by sequence modification, demonstrating their reduced antigenicity when tested against sera from subjects who developed ADAs against the original biotherapeutic [105,106,107]. B-cell epitope removal has also shown promise in promoting tolerance to autoantigens such as the nicotinic acetylcholine receptor [108]. Finally, anti-idiotype antibodies may be problematic if they neutralize the activity of a therapeutic monoclonal antibody but, conversely, anti-idiotypes may be developed deliberately in some cases, e.g., by phage display, and employed to block neutralizing polyclonal antibodies that patients develop against therapeutic proteins [109].

Clinically significant anti-FVIII ADAs are class-switched, predominantly comprised of IgG1 and IgG4, and the detection of IgG4 and/or of higher-affinity FVIII-specific antibodies has been proposed as a predictive biomarker of a developing inhibitor response [110,111,112]. The identification of earlier prognostic biomarkers, e.g., specific cytokine, endosome or cellular signatures, is as yet an unrealized goal and a subject of current research efforts. Such early detection could improve patient outcomes, as clinical Immune Tolerance Induction protocols have higher success rates when initiated while antibody titers are still relatively low.

**Figure 2:**
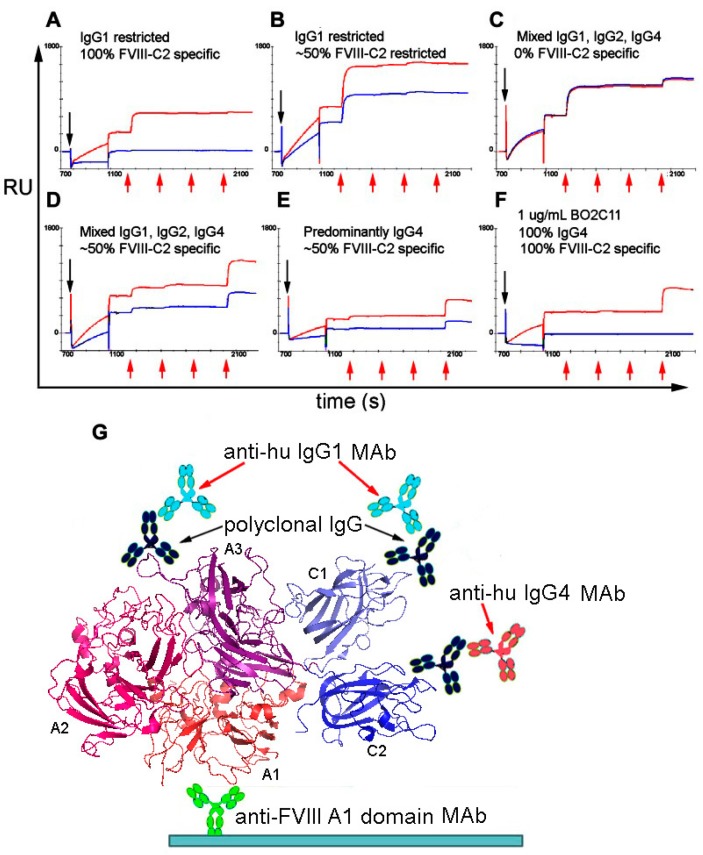
“Saturation SPR” method to characterize polyclonal anti-FVIII anti-drug antibodies (ADAs) in patient plasma samples [114]. First a high-affinity MAb specific for the FVIII A1 domain is covalently immobilized on the biosensor surface, and FVIII protein is then injected and captured by this MAb (0–700 s, not shown in figure). A plasma sample (pre-treated with caprylic acid to minimize biosensor fouling) is injected next (black arrows) followed by sequential injections of anti-hu IgG1, IgG2, IgG3 and IgG4 MAbs (red arrows), all at saturating concentrations. The vertical displacements (in RUs) may be converted to total mass at the biosensor surface following each injection, which is then converted to the total bound antibody concentration assuming an average molecular weight of 150 kDa. (**A**–**F**): The red and blue sensorgram curves depict matched plasma samples pre-incubated with (red) and without (blue) saturating (1 µM) recombinant FVIII-C2 domain protein, which will compete with the polyclonal anti-FVIII-C2 antibodies in the plasma samples. Thus, these paired SPR experiments indicate the total anti-FVIII polyclonal antibody titer, the fractions of IgG1, IgG2, IgG3 and IgG4 in the samples, and the fraction of antibodies specific for the FVIII C2 domain. Panels A-E illustrate the diverse phenotypes of the anti-FVIII IgG response in patients with allo-or autoimmune anti-FVIII antibodies. Panel F is a control experiment in which a patient-derived IgG4 monoclonal antibody specific for the FVIII C2 domain is tested in the same assay. No patient samples contained FVIII-specific IgM or IgGA (not shown). (**G**) The biosensor assay format is shown schematically, with the FVIII crystal structure [116] in ribbon representation.

Sandwich ELISA assays and competitive ELISAs have been used to define isotypes, subclasses and apparent/relative affinities of anti-FVIII antibodies in HA inhibitor patient samples [110]. The proportions of FVIII-specific immunoglobulin isotypes in patient samples have been analyzed semi-quantitatively by ELISAs employing isotype-specific MAbs, together with engineered MAbs consisting of the variable heavy chain domain of a FVIII-specific scFv fused to the constant CH1-CH2-CH3 fragments of IgG1, IgG2, IgG3 and IgG4 as internal controls [113]. As an alternative method, Lewis et al. developed a novel SPR-based assay to quantify both the total anti-FVIII antibody titers, and the amount of each subclass comprising this total [114], exploiting the fact that the change in Response Units (RUs) upon saturation binding of an antibody to a ligand immobilized on a biosensor chip is easily converted to the total mass of the captured antibodies (Figure 2) [115]. The molecular weight of monoclonal or polyclonal IgG is ~150 kDa. Therefore, the number of IgG molecules captured by an immobilized antigen such as FVIII can be estimated. Similarly, the RU increases following subsequent injections of anti-IgG1, anti-IgG2, anti-IgG3 and anti-IgG4 MAbs can be converted to the numbers of these molecules immobilized, thereby yielding the total amount and relative proportions of each antibody isotype. This assay can be used to characterize either purified polyclonal antibody or plasma/serum samples (pretreated with caprylic acid to reduce nonspecific binding) to acceptable levels. It could easily be adapted to phenotype antibodies against other biotherapeutics.

Linear B-cell epitopes on allo-or auto-antigens may be identified by incubating plasma or serum samples in wells of microarrays containing immobilized peptides corresponding to the protein antigen of interest [117]. Typically, 15- to 20-mer synthetic peptides, with overlapping sequences spanning the biotherapeutic protein sequence, are attached covalently, via a flexible linker to avoid steric interference, to a solid surface, e.g., derivatized glass slides. Despite several caveats, including the inability of peptides to recapitulate conformational epitopes comprised of non-contiguous segments of a protein amino acid sequence; lower affinities than protein binding interactions; and false negatives and positives, such microarrays can yield valuable information regarding clinically relevant immune responses. Some of the reactive peptides in fact correspond to “loops” on the protein’s surface, correctly identifying at least part of the corresponding B-cell epitope. Assays of cross-sectional clinical samples can identify patterns associated with disease states, and longitudinal samples from the same subject can illustrate epitope drift during the course of treatment, e.g., immunotherapies following antibody detection. Such array analyses have proven valuable in vaccine studies [118,119] and in profiling of autoimmune disorders, infections and cancer [117], and they may also be applied to characterize ADAs [120]. Some promising recent developments include combination of peptide array data with results of phage display, protein structural information, B-cell epitope prediction algorithms, etc. [121]. Use of microarrays consisting of immobilized proteins, e.g., auto- or allo-antigens, is an emerging proteomics approach to characterize pathologic antibodies, potentially yielding information characterizing conformational epitopes that is complementary to the finer mapping of epitopes achievable with peptide microarrays [122,123,124]. The use of proteins instead of peptides as capture molecules in assays of clinical samples has associated challenges, e.g., relating to protein stability, sensitivity and reproducibility of assays using proteins spotted in microarray wells, etc., but ongoing technical advances indicate that such proteomics analyses will increasingly play a role in clinical diagnostics and epitope mapping applications [125].

Phage display technology may be used to detect ADAs and to map linear or conformational epitopes recognized by ADAs, MAbs, etc. [126,127]. The use of phage display libraries to characterize immune responses in studies of vaccines [128,129,130,131] allergy [121,127,132] and autoimmunity [133] is highly analogous to profiling of alloimmune responses to a biotherapeutic. Phage display methodology is continually evolving and improving, particularly by incorporating additional techniques including bioinformatics and structural analysis for quality control [121,127,134,135,136] and to identify clusters of surface-exposed side chains on protein antigens that recapitulate the patterns/properties of the phage-displayed proteins (e.g., scFvs) or peptides [133].

Prediction of B-cell epitopes on protein antigens appears to be even more challenging than prediction (without significant over-prediction) of T-cell epitopes, because B-cell epitopes are generally conformational, i.e., comprised of non-contiguous rather than linear amino acid sequences, and the three-dimensional structure of the antigen is often unknown. Significant creative effort is going into development of computational methods to identify and modify B-cell epitopes to reduce antigenicity, e.g., with recent application to recombinant arginine deiminase (a cancer biotherapeutic) [137]. Incorporation of some experimental data, e.g., peptide array or phage-display results, into epitope identification approaches greatly improves success rates, compared to ab initio methods based on amino acid sequence propensities to form beta turns, or machine-learning based algorithms [42]. When considering B-cell epitope modification to reduce antigenicity, reliable, experimental determination of the B-cell epitopes is therefore preferred when possible.

## 6. Prediction of Immunogenicity and Patient Outcomes

In addition to characterizing the ADAs themselves, immunoprofiling of patient plasma samples has demonstrated differences in circulating cytokines and in types of microparticles between naïve HA subjects, age-matched healthy controls [138] and HA subjects with and without a neutralizing ADA response [139,140,141,142,143]. However, none of these differences has yet been incorporated into general clinical practice or shown to be predictive, as the studies have been primarily cross-sectional. Similarly, the need for standardized assays utilizing predictive biomarkers of ADAs against any biologics to improve clinical decision-making has been pointed out [144]. Intuitively, one would expect that such biomarkers may be discovered sooner in the context of alloimmune responses to therapeutic MAbs or protein replacement therapies for monogenic diseases such as the hemophilias, compared to autoimmune disorders, because both the offending antigen and the doses and timeline of its administration are known precisely. Longitudinal studies in which samples are collected from patients and relevant animal models will be essential for profiling the naïve immune response to the biotherapeutic and the subsequent maturing immune response resulting in clinically significant ADAs versus non-responsiveness. The identification of reliable predictive biomarkers would allow rational, evidence-based criteria for deciding which patients are likely to benefit from (and accept the associated risk of) transient immunosuppression therapies during biotherapeutic administration.

## 7. Summary

This non-comprehensive review focuses on several current approaches, dilemmas and novel techniques to profile ADA responses and mitigate their impact on the translation of promising biotherapeutics. I have focused here on studies of clinical samples obtained from patients and normal healthy controls, rather than animal model studies, in part because immunogenicity in humans is often not predicted by animal testing. In particular, the impact of engineering therapeutic proteins (e.g., changing their amino acid sequence to modify a specific property) is best evaluated by *in silico* predictions and direct assays of cellular fractions isolated from blood samples; however, even with extensive testing, immunogenicity may not be detected until a clinical trial is under way. Animal models, including mice with humanized immune systems, are invaluable for mechanistic studies, including testing of novel methods to induce tolerance to biotherapeutics. A central question, relevant to studies of both immunogenicity and tolerance, is why most patients exposed to biotherapeutics such as FVIII in fact develop peripheral tolerance, whereas others develop ADAs that may preclude further treatment with the immunogenic drug. Significant effort has gone into attempts to “de-immunize” several therapeutic proteins, and to characterize the ADA response at both the cellular and antibody level. Protein engineering strategies and clinical protocols to induce tolerance are complementary approaches to improve patient outcomes. Prospective studies of naïve patients during their initial exposures to a biotherapeutic can provide particularly important mechanistic information. Especially in the case of rare disorders such as hemophilia A and B, patient numbers and available samples are limited. Therefore, creative partnerships between those conducting pharmaceutical or government-funded clinical trials of biotherapeutics, and bench scientists carrying out basic science studies, hold promise to identify predictive biomarkers of immunogenicity and to suggest novel potential tolerogenic therapies of the future [77,78,79,145,146,147,148,149,150].

## 8. Patents

The author is an inventor on patents regarding Factor VIII immunogenicity.
